# Antimicrobial Prescribing Patterns in GP Practices in Northern Ireland

**DOI:** 10.3390/antibiotics13111050

**Published:** 2024-11-05

**Authors:** Heather M. Coleman, Eimear Clifford, Kingston Rajiah, Nermeen Ali, Aaron Courtenay, Deborah Lowry, Iain G. Jack, Ahmed Abuelhana

**Affiliations:** School of Pharmacy and Pharmaceutical Sciences, Ulster University, Coleraine, BT 52 1SA UK; eimear.clifford@hotmail.co.uk (E.C.); k.rajiah@ulster.ac.uk (K.R.); n.ali@ulster.ac.uk (N.A.); a.courtenay@ulster.ac.uk (A.C.); d.lowry@ulster.ac.uk (D.L.); i.jack@ulster.ac.uk (I.G.J.); a.abuelhana@ulster.ac.uk (A.A.)

**Keywords:** antibiotics, antimicrobial prescribing, antimicrobial resistance, antimicrobial stewardship, AWaRe, COVID-19, general practice, general practice pharmacists, primary care

## Abstract

**Introduction:** Antimicrobial resistance (AMR) is a global health threat requiring immediate attention as it is set to cause ten million deaths worldwide by 2050, overtaking that of cancer. Continuation of overuse and/or misuse of these crucial medicines will prevent future generations from reaping the benefits, as the pandemic of AMR spirals out of control. **Aims:** The primary aim of this study was to investigate antimicrobial prescribing patterns in General Practices throughout Northern Ireland. A secondary aim was to analyse the impact of the COVID-19 pandemic on antimicrobial prescribing and consumption patterns in GP practices in Northern Ireland. **Methods:** A retrospective, cross-sectional quantitative study was designed to measure, analyse, and evaluate the antimicrobial prescribing patterns within GP practices in Northern Ireland, using open access Business Services Organisation (BSO) data. **Results:** A total of 3,168.78 kg of antibacterial drugs were prescribed in primary care throughout the duration of the study. Penicillins were the most prescribed class (59.79%), followed by tetracyclines (10.68%) and macrolides (9.53%). Access group antibiotics were the most frequently prescribed (79.35%), followed by Watch group antibiotics (20.64%), with Reserve group antibiotics equating to nearly 0% despite being prescribed. The Derry GP Federation prescribed and dispensed the greatest amount of antibiotics overall in Northern Ireland (10.90%). Despite there being no significant difference in antibiotic prescribing amongst GP federations prior to and during the COVID-19 pandemic (unpaired *t*-test, *p* > 0.05), there were differences in prescribing of individual drug classes throughout this period. **Conclusions:** Despite meeting World Health Organisation (WHO) targets, GP practices within Northern Ireland must achieve more to further reduce antimicrobial consumption. Although antibiotic prescribing rates here are on the decline, there was no significant difference in prescribing amongst GP federations pre- and midst-COVID-19 pandemic, thus sufficient strategies such as increased communication between colleagues and supportive measures must be implemented within GP practices to enhance antimicrobial stewardship (AMS) across Northern Ireland.

## 1. Introduction

Antimicrobial resistance (AMR) is a global threat posing a significant challenge to healthcare systems across all nations. International collaboration is needed to effectively manage growing needs and to implement clear strategies to manage AMR [1]. As of 2019, over 1.2 million deaths worldwide were due to AMR [2]; however, by 2050 it is estimated that this figure will rise to 10 million—that is one person every three seconds [3]. Regardless of economic status, AMR affects countries worldwide, resulting in increased treatment costs for resistant infections due to the need for reserved therapies. Therefore, if AMR fails to be addressed globally, it will have detrimental effects directly on patient care, but additionally on healthcare systems and subsequent economies [4]. Antibiotics when used appropriately are effective antibacterial agents; however, they are not universally effective against all types of bacteria. Those antibiotics that acquire resistance to bacteria over time develop what is known as extrinsic resistance [5]—a considerable problem due to the lack of discovery of novel antibiotic agents since the late 1970s, despite prediction by Alexander Fleming [6]. When first discovered in the 1920s, antimicrobials were revolutionary to healthcare, benefitting surgery through the provision of chemo-prophylaxis, aiding in the treatment of common infections, as well as reducing the risks associated with childbirth [3]. Nonetheless, with increasing AMR, treatment of infection is becoming progressively challenging. Previously routine surgical procedures are becoming increasingly risky owing to complications in treating post-operative infections, and thus mortality rates are increasing [5]. If overuse and misuse of these so-called ‘Wonder Drugs’ remain, bacteria will continue to acquire resistance, causing the future of society to revert to times without such life-saving treatments [7]. Common infections, such as TB, pneumonia, and gonorrhoea, are becoming increasingly difficult to treat due to the effects that both overprescribing and increased consumption of antibiotics have on AMR [8]. Despite a 19.1% reduction in antibacterial prescribing in Northern Ireland (NI) between 2012 and 2019, NI’s antibacterial prescribing rates remain the highest in the UK [9]. Even with the development of novel antibiotics, these too would become resistant without fundamental changes in prescribing behaviour. Therefore, behavioural changes need to be implemented throughout healthcare if there is hope of diminishing AMR [8].

**Figure 1:**
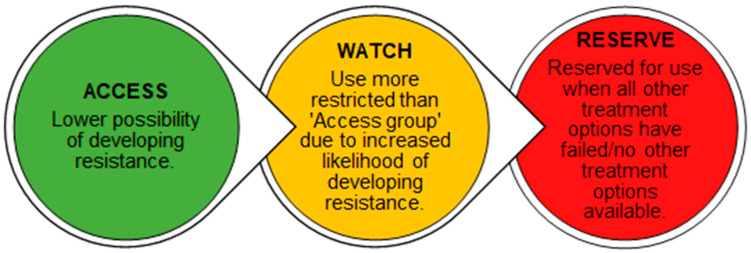
The WHO AWaRe Classification of Antibiotics [6].

As Northern Ireland’s antimicrobial prescribing and consumption are markedly higher than that of England [5], the NI Department of Health Strategy for Tackling Antimicrobial Resistance (STAR) [10], was replaced by the One Health Joint Plan of Action (OH JPA) 2022–2026, to encompass animal, environmental, and human health, as a more holistic approach to AMR [9]. In addition, the Strategic Antimicrobial Resistance and Healthcare-Associated Infection (SAMRHAI) group have the responsibility of ensuring behaviours to reduce antimicrobial consumption are implemented across Northern Ireland, in an attempt to decrease AMR, and thus lower antimicrobial-resistant infections throughout Northern Ireland [6]. To enhance antimicrobial stewardship (AMS) worldwide, the World Health Organisation (WHO) Expert Committee developed the AWaRe Classification of antibiotics, allocating each antibiotic into one of three groups; Access, Watch, and Reserve (Figure 1) [11]. The WHO has supported antimicrobial surveillance and AMS programmes worldwide, addressing the urgency of AMR [12]. Their 13th General Programme of Work set a target for all countries that, by 2023, a minimum of 60% of the antimicrobials consumed should be Access group antibiotics [13]. In addition, a primary objective of “Changing the Culture 2019–2024—One Health, Five-year Action Plan on Antimicrobial Resistance” in NI is, by 2024, to decrease drug-resistant infections by 10%, and to reduce antimicrobial consumption in humans by 15% [14]. Other efforts to reduce AMR include those of primary care in NI, where General Practice Pharmacists (GPPs) have been employed since 2015, with the crucial role of enhancing AMS amongst prescribers [9]. Currently, there are over three hundred and seventy GPPs working within GP teams across Northern Ireland to enhance AMS via prescribing practices throughout primary care [15].

### COVID-19 Pandemic

The COVID-19 pandemic has had a major impact worldwide [16]. In the UK alone, COVID-19 has affected more than 20 million d people and caused almost 2000 deaths [17]. Changes implemented throughout healthcare, including those to frameworks, guidelines, and access to care, were to reduce transmission and control infection. However, these were likely to impact and negatively affect AMR [18]. Prior to the COVID-19 pandemic, antibiotics were inappropriately overprescribed in primary care England for viral infections such as influenza [19]. Thus, a key focus was on AMS, to reduce consumption of antibiotics, reduce AMR, and thus subsequent infections [20]. Nonetheless, as it was difficult to differentiate between COVID-19 and bacterial pneumonia early in the pandemic, as well as the lack of effective antivirals available, patients received unnecessary antibiotics [21]. A study in North-West London found that although overall antibiotic prescribing decreased from the first lockdown in 2020, possibly due to social distancing measures [22], those hospitals with increased numbers of COVID-19 patients had higher rates of antibiotic prescribing—regardless of the low rate of bacterial co-infection [23].

Remote consultations, reduced access to antimicrobial susceptibility testing (AST), and lack of COVID-19 testing in the beginning challenged the appropriateness of antibiotic use; however, there is currently limited evidence available to support this [24]. Moreover, although prior to COVID-19 AMR required global, immediate action, since then it is even more so of importance to address [18].

To the best of the investigator’s knowledge, very limited studies have explored antimicrobial prescribing patterns in General Practices throughout Northern Ireland. The lack of literature surrounding Northern Ireland specifically highlighted the imperative need for conducting this study, aiming to bridge the existing gap in knowledge and gain a deeper understanding of both prescribing and resistance patterns in primary care in Northern Ireland. It is therefore crucial that sufficient research is undertaken to comprehend antimicrobial prescribing patterns here, and thus aid reduction in antimicrobial consumption and resistance by enhancing AMS within primary care.

The overall aim of this study was to analyse antimicrobial prescribing patterns in General Practices across Northern Ireland. A secondary aim was to investigate the impact of the COVID-19 pandemic on antimicrobial prescribing and consumption patterns in GP practices in Northern Ireland.

Specific objectives include the following:

- Explore the current literature on antimicrobial prescribing patterns in primary care as a base for understanding the research;
- Investigate the total antibiotics prescribed for the duration of the study and compare these to the WHO’s AWaRe classification of antibiotics and WHO prescribing targets;
- Assess antibacterial prescribing patterns amongst GP federations in Northern Ireland and compare prescribing patterns of the three most prescribed antibiotic classes between the federations;
- Analyse antibiotic prescribing patterns within a defined period of time pre- and during COVID-19; to investigate antibiotic prescribing and consumption prior to and during the COVID-19 pandemic.

## 2. Results

### 2.1. Antimicrobial Prescribing Patterns in GP Surgeries Across Northern Ireland

#### 2.1.1. Number of Antibiotics Prescribed

Antimicrobial prescribing patterns within 330 GP surgeries in April 2019, and 325 GP surgeries in November and December 2019 across Northern Ireland were analysed via Business Services Organisation (BSO) open access data.

During this time, a total of 3,168.78 kg of antibacterial drugs were prescribed—either by a GP, nurse, or other non-medical prescriber linked to a GP practice—and dispensed in Northern Ireland; 942,258.93 g (30%), 1,074,084.44 g (34%), and 1,152,432.01 g (36%) were prescribed and dispensed during April, November, and December 2019, respectively (Table 1). The total amount of antibiotics prescribed during the study period equates to approximately 1658.61 g per 1000 inhabitants when calculated against Northern Ireland’s population size of 1,910,500.

**Figure 2:**
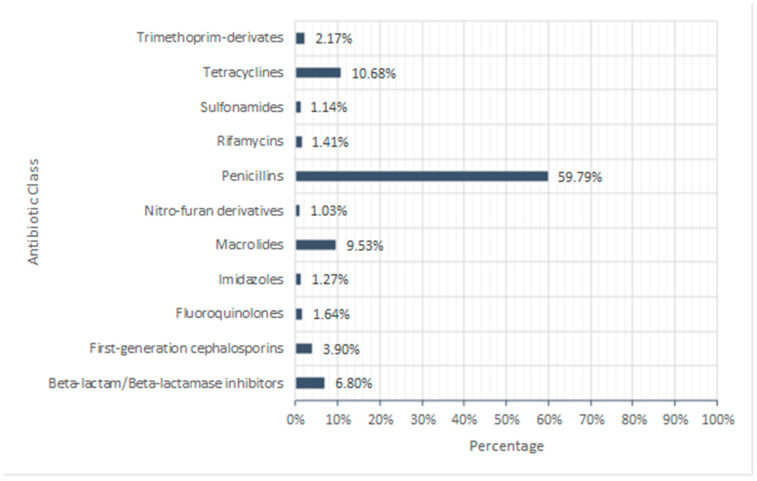
Percentage of antibiotic class prescribed in GP surgeries across Northern Ireland throughout the duration of the study (Those antibiotic classes prescribed < 0.25% were excluded from the figure (see Table 1)).

In total, 26 different antibacterial classes were prescribed and dispensed during this period, of which penicillins were the most prescribed class (59.79%), followed by tetracyclines (10.68%) and macrolides (9.53%) *(*Figure 2). Aminoglycosides, amphenicols, antimycobacterial, beta-lactam/beta-lactamase inhibitors antipseudomonal, carbapenems, fusidane, glycopeptides, lincosamides, oxazolidinones, phosphonics, rifamycins with antimycobacterial, second-generation cephalosporins, steroid antibiotics, sulfones, and third-generation cephalosporins each contributed less than one per cent to the total amount of antibiotics prescribed during this period (Table 1). Beta-lactam/Beta-lactamase inhibitors contributed 6.80%, first-generation cephalosporins contributed 3.90%, trimethoprim-derivatives contributed 2.17%, fluoroquinolones contributed 1.64%, rifamycin’s contributed 1.41%, imidazole’s contributed 1.27%, sulfonamides contributed 1.14%, and nitro-furan derivatives contributed 1.03% towards the total amount of antibiotics prescribed and dispensed.

#### 2.1.2. Number of WHO AWaRe Classification Antibiotics Prescribed

Of the 3,168,775.38 g of antibacterial drugs prescribed and dispensed, 3,158,944.33 g (99.69%) was classified according to the WHO’s AWaRe Classification of antibiotics, according to their likelihood of developing resistance. Those antibacterial drugs remaining did not fall within the Access, Watch or Reserve groups according to the WHO’s classification. Dapsone, ethambutol, isoniazid, pyrazinamide, rifampicin with isoniazid, and sodium fusidate did not appear in the AWaRe Classification of antibiotics. Additionally, co-fluampicil is stated as ‘not recommended’ by the WHO. As shown in Table 2, Access group antibiotics were the most frequently prescribed, totalling 2,506,756.21 g (79.35%), succeeded by Watch group antibiotics with a total of 652,113.72 g (20.64%), and finally Reserve group antibiotics consisting of only 74.40 g, equating to approximately 0.00%.

### 2.2. Antimicrobial Prescribing Amongst GP Federations in Northern Ireland

**Figure 3:**
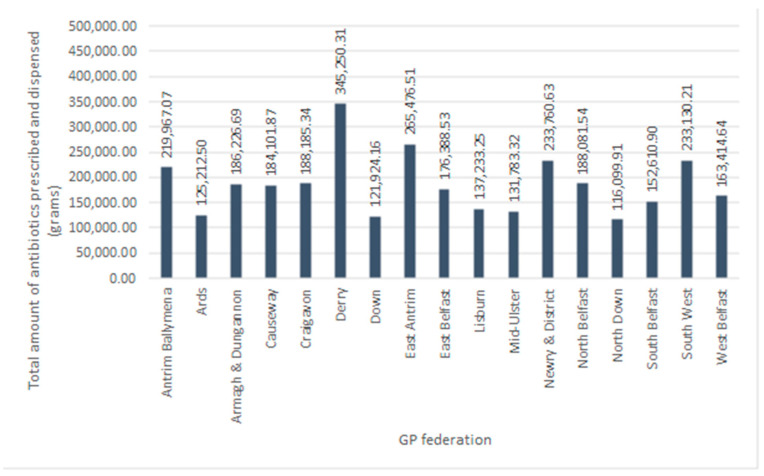
Total amount of antibiotics prescribed and dispensed across all GP federations in Northern Ireland, throughout the duration of the study.

**Figure 4:**
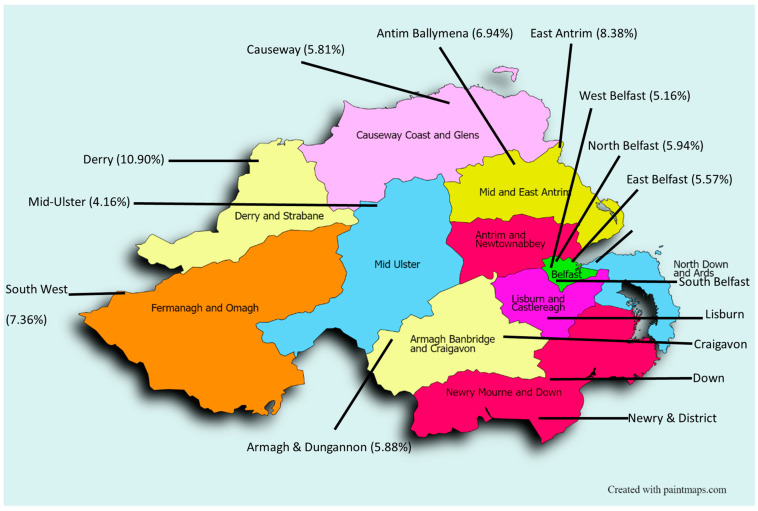
Total amount of penicillins, tetracyclines, and macrolides prescribed and dispensed in GP Federations across Northern Ireland throughout the duration of the study.

Analysis of antibacterial prescribing patterns amongst the seventeen GP federations (*n* = 17) in Northern Ireland, through April, November, and December 2019, showed the amount (g) of antibacterial drugs prescribed followed a normal distribution (Kolmogorov–Smirnov test, *p* > 0.05), with a mean of 186,402.79 g (±7.78%). There was a statistically significant difference between the total amount (g) of antibiotics prescribed between GP federations (One-sample *t*-test, *p* < 0.05). During this time, Derry GP Federation prescribed and dispensed the greatest amount of antibiotics, almost 11% (Figure 3). Figure 4 shows the relative percentage of antibacterials prescribed in each of the 17 GP Ffederations; Antrim Ballymena, Armagh and Dungannon, Causeway, Craigavon, East Antrim, East Belfast, Newry and District, North Belfast, South West, and West Belfast GP Federations contributed between five and ten percent to the total amount of antibacterials prescribed amongst the GP federations. Ards, Down, Lisburn, Mid-Ulster, North Down, and South Belfast GP federations contributed less than five percent to the total antibacterial drugs prescribed throughout the duration of the study.

**Figure 5:**
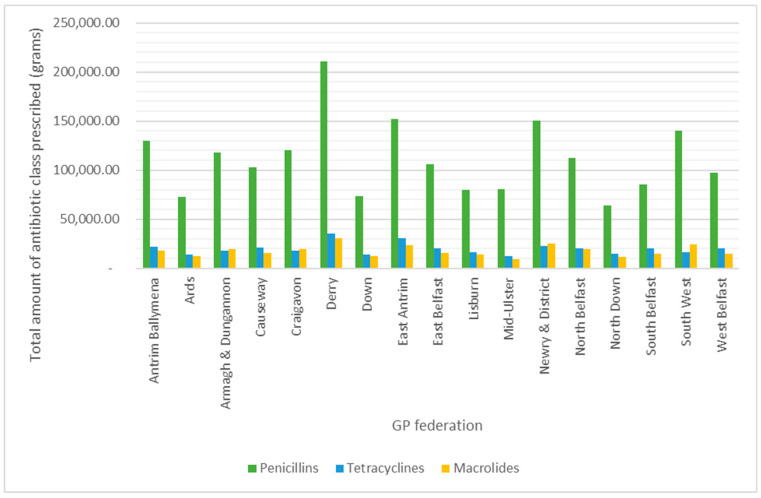
Percentage of antibiotics prescribed and dispensed across all GP federations in Northern Ireland throughout the duration of the study.

The three most prescribed antibiotic classes through April, November, and December 2019 were penicillins (59.79%), tetracyclines (10.68%), and macrolides (9.53%) (Figure 2). Analysis of these antibiotic classes across the GP federations (Table 3) showed the amount of each class prescribed followed a normal distribution (Kolmogorov–Smirnov test, *p* > 0.05), with respective means of 111,452.07 g (±8.15%), 19,902.85 g (±7.19%), and 17,759.31 g (±7.71%) for penicillins, tetracyclines, and macrolides. A comparison of the amount (g) prescribed of these three major antibiotic classes across GP federations showed there was a statistically significant difference in antibiotic prescribing between GP federations (Two-way ANOVA, *p* < 0.05). Once again, the Derry GP Federation prescribed and dispensed the greatest number of grams of penicillins, tetracyclines, and macrolides (Figure 5); 210,782.50 g (11.12%), 35,460.46 g (10.48%), and 30,704.76 g (10.17%), respectively.

### 2.3. Antibiotic Prescribing Patterns Pre- and Midst-COVID-19 Pandemic

**Figure 6:**
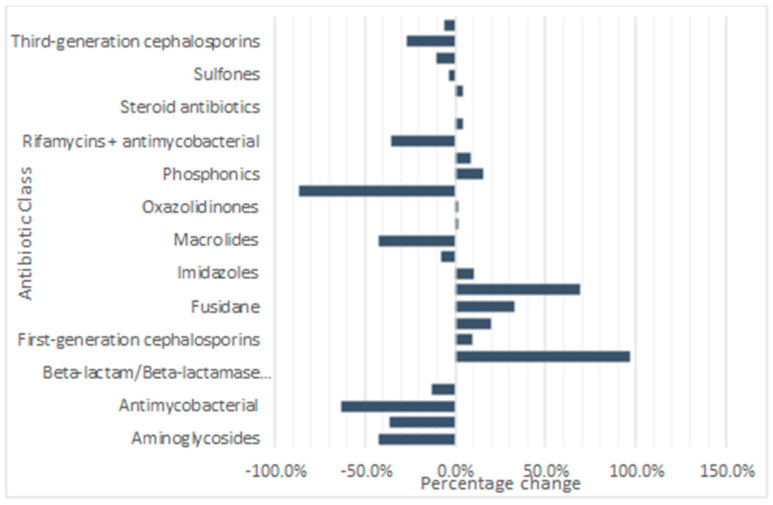
Percentage change in antibiotic classes prescribed and dispensed pre- and midst-COVID-19 pandemic.

Antibiotic prescribing patterns pre-COVID-19 pandemic (April, November, and December 2019) and during the COVID-19 pandemic (November 2020, December 2020, and January 2021) were investigated. Analysis showed that the data for pre- and midst-COVID-19 pandemic were close to normality (Kolmogorov–Smirnov test, *p* < 0.001). However, when the data were log transformed, it was found to be normally distributed (Kolmogorov–Smirnov test, *p* > 0.05). There was no statistically significant difference between antibiotic prescribing pre-COVID-19 pandemic when compared to midst-COVID-19 pandemic (Unpaired t-test, *p* > 0.05, *n* = 26). Nonetheless, there were differences in prescribing patterns within each class of antibiotics throughout the duration of the study (Figure 6).

Carbapenems (+96.88%), first-generation cephalosporins (+9.49%), fluoroquinolones (+19.88%), fusidane antibiotics (+32.80%), glycopeptides (+69.34%), imidazoles (+10.37%), nitro-furan derivatives (+1.25%), oxazolidinones (+1.59%), phosphonics (+15.63%), rifamycins (+8.78%), second-generation cephalosporins (+4.01%), and sulfonamides (+4.27%) increased in prescribing during the COVID-19 pandemic when compared to the amount prescribed prior to the COVID-19 pandemic. Aminoglycosides (−42.12%), amphenicols (−36.36%), antimycobacterial (−63.18%), beta-lactam/beta-lactamase inhibitors (−13.49%), beta-lactam/beta-lactamase inhibitors antipseudomonal, lincosamides (−7.49%), macrolides (−42.16%), penicillins (−86.37%), rifamycins with antimycobacterial (−35.78%), steroid antibiotics, sulfones (−3.79%), tetracyclines (−10.63%), third generation cephalosporins (−26.78%), and trimethoprim derivatives (−6.33%) decreased in prescribing during the COVID-19 pandemic in comparison to the amount prescribed prior to the COVID-19 pandemic. Additionally, no beta-lactam/beta-lactamase inhibitors antipseudomonal or steroid antibiotics were prescribed and dispensed during the COVID-19 pandemic.

## 3. Discussion

### 3.1. Number of Antibiotics Prescribed

The overall aim of this study was to analyse antimicrobial prescribing patterns in General Practices across Northern Ireland. This was achieved via an analysis of BSO open access data in 330 GP Practices in April 2019, and 325 GP Practices in November and December 2019. The reduction in GP surgery numbers was due to GP surgery closures and/or merges. April, November, and December 2019 represented a period prior to the COVID-19 pandemic, and thus was indicative of day-to-day standard practice within GP surgeries. April was included as a control month outside of the winter season, where antibacterial prescribing naturally increases due to winter illnesses [25,26].

The total amount of antibiotics prescribed and dispensed during the study was 3,168,775.38 g. This includes those prescribed by a GP, nurse, or other non-medical prescriber linked to a GP practice, such as a pharmacist independent prescriber; however, it does not include hospice items or private prescriptions. Additionally, all prescribed antibiotics in the data set were dispensed from a community pharmacy; otherwise, they were not included in the BSO open access data [27]. Studies conducted in other countries often express antimicrobial prescribing rates using the metric of Defined Daily Dose (DDD). However, access to such data for Northern Ireland was restricted, presenting a challenge in comparing antibacterial prescribing rates in Northern Ireland with those in other parts of the world. Nonetheless, in relation to comparator countries, such as the Netherlands, Japan, and China, the amount of antibiotics prescribed within primary care in Northern Ireland remains above average, despite a decrease in prescribing in 2019; its antibacterial prescribing is almost double that of the Netherlands [28]. Furthermore, within the UK, Northern Ireland has the highest antimicrobial prescribing rates, despite a reduction of 19.1% between 2012 and 2019 [9]. Although antimicrobial prescribing in Northern Ireland is declining, it remains a work in progress when considering its population, 1,903,175 people [29] (compared to the Netherlands, which has a population of 17,943,348 people [30]). Although Northern Ireland has a much smaller population than the Netherlands, it surpasses the Netherlands in antimicrobial prescribing rates among other European countries [31].

Of the antibiotics prescribed throughout the duration of the study, penicillins (59.79%), tetracyclines (10.68%), and macrolides (9.53%) were the most frequently prescribed classes of antibiotics in Northern Ireland. This is consistent with a previous study conducted in primary care in England where penicillins accounted for 48.8%, macrolides 13.4%, and tetracyclines 12.4% of antibiotic prescriptions [32]. Similarities in prescribing behaviours, treatment guidelines, and patterns of disease between Northern Ireland and England may explain why the UK’s antibacterial prescribing is above the global average [28]. This study focused on overall antibiotic consumption in Northern Ireland, unlike previous studies which looked further at antibiotic prescribing in terms of socio-demographic factors such as gender, age, and location [33]. It is therefore evident that more in-depth research is required to fully understand the reasoning behind the high level of antimicrobial prescribing rates in Northern Ireland and its significant contribution to AMR.

### 3.2. Number of WHO AWaRe Classification Antibiotics Prescribed

Classification of the antibiotics prescribed and dispensed throughout the study according to the WHO’s AWaRe Classification of antibiotics revealed that nearly 80% of antibacterials were Access group antibiotics. This is a positive outcome as the WHO 13th General Programme of Work 2019–2023 recommends that at least 60% of antimicrobials consumed in each country should be Access group antibiotics [11]. Therefore, Northern Ireland met this target by 2023, indicating the use of optimal empiric therapies for the treatment of common bacterial infections. Despite this, antibacterial drugs not included in the AWaRe Classification of antibiotics, as well as those not recommended by WHO were prescribed during the specified period of data collection. Watch and Reserve antibiotics accounted for just over 20% of the antibiotics prescribed during the study. Reserve list antibiotics were considered neglected as they contributed nearly 0% to the overall prescriptions. This contribution is not statistically significant, despite their inclusion. Such drugs are not recommended for use in clinical practice and may worsen the already existing pandemic of AMR if they continue to be prescribed. Overall, this may hinder the primary objective of “Changing the Culture 2019–2024—One Health, Five-year Action Plan on Antimicrobial Resistance” which employs an integrated approach involving government, professionals, research, business, and the wider population to combat AMR. However, its primary objective to reduce antimicrobial consumption in humans by 15% and to lower drug-resistant infections by 10% by 2024 [14], may not be reached if overuse and misuse of antimicrobials remain, and thus will exacerbate AMR.

GP federations are led and funded by GPs in Northern Ireland, with the aim of providing more efficient, higher-quality care for patients by implementing strategies together to better suit local needs through the development of relevant patient services [34]. Analysis of antimicrobial prescribing patterns between the seventeen GP federations in Northern Ireland showed that there was a statistically significant difference between the total amount of antibiotics prescribed amongst these federations. The Derry GP Federation prescribed the highest amount of antibiotics in Northern Ireland, at almost 11%, followed by the East Antrim GP Federation (8.38%). Further investigation into the prescribing of penicillins, tetracyclines, and macrolides confirmed that the Derry GP Federation not only prescribed the highest amount of antibiotics, but also consistently prescribed the highest proportions of penicillins (11.12%), tetracyclines (10.48%), and macrolides (10.17%) among the federations.

It was expected that a greater number of GP practices within a federation would result in a higher amount of antibiotics prescribed. However, this was not the case. The Derry GP Federation consisting of 27 GP practices, prescribed a higher amount of antibiotics than expected. In contrast, Newry and District GP Federation with 29 GP practices contributed 7.38% to the total antibiotic prescribing in Northern Ireland. This indicates that other factors must be taken into consideration when comparing prescribing patterns between the GP federations. One such factor would be the number of GPPs employed within each federation. GPPs have been part of the GP practice teams across Northern Ireland for the past eight years, primarily to reduce antimicrobial consumption and resistance [35]. The Derry GP Federation employs the equivalent of 28.5 full-time practice-based pharmacists [36], while Newry and District employs 31 [37]. The enhanced role of the pharmacist within the GP practices may explain the lower antibiotic prescribing in the Newry and District federation as here they exist in greater numbers. The roles and responsibilities of GPPs in checking the appropriateness of prescriptions, enhancing AMS within primary care, as well as educating both the public and other healthcare professionals within the GP practice team, have been well described [38,39] which has clearly had a positive impact on antibiotic consumption throughout Newry and District GP Federation, and thus its contribution to AMR. This is consistent with a previous study in which the collaboration of GPs with GPPs was shown to reduce antimicrobial prescribing by 16.09% [40]. This displays the importance of the inclusion of GPPs within GP practice teams, suggesting that a possible approach in which the Derry GP Federation could reduce its antibiotic consumption would be through employing more GPPs within their GP practice teams, to enhance AMS within the practice in order to address the growing challenge of AMR.

Another fundamental aspect that should be considered is the level of deprivation across geographical areas within each of the GP federations. A previous study demonstrated that those GP practices located in more deprived areas of Northern Ireland had a higher rate of antibiotic prescribing [41]. Considering Foyle (Derry) is one of the most deprived assembly areas overall in Northern Ireland [42], the findings of this research align with the previous study as the Derry GP Federation falls geographically within the Foyle assembly. Earlier research concluded that GP practices with high numbers of patients aged over 65 years had increased antibiotic prescribing rates compared to other GP practices [43]. However, this is not consistent with the findings of this study as Foyle assembly is in fact one of the areas in Northern Ireland with the smallest proportion of older people (> 65 years) in the population [42]. Therefore, increased antimicrobial prescribing observed in the Derry GP Federation may be attributed to various factors, including income, living conditions and education.

Furthermore, it has been previously concluded that multiple factors within the GP practice itself influence antibiotic prescribing in primary care, including a combination of the GP’s experience and confidence in clinical decision making [44]. The challenge of balancing patient expectations with clinical decision making creates friction in the GP-patient relationship [45] leading to difficulties in shared decision making [46]. Efforts such as deferred antibiotic prescribing have not significantly reduced antibiotic consumption, as patients often collect antibiotics immediately, regardless of the GP’s advice [47]. GP practices with low antibiotic prescribing attribute their success to consistency amongst practitioners within the practice, improved communication with colleagues, and supportive practice policies within the surgery [44]. On the other hand, GP practices with high antibiotic prescribing rates report needing support when not providing antibiotics, as well as longer appointment times for patient consultations [48]. With the current knowledge, potential solutions for those GP practices included in this study with high antibiotic prescribing rates, such as those within the Derry GP Federation, can be proposed. Increasing support, resources, and consistency within practices, along with enhanced patient-centred communication could help decrease antibiotic prescribing rates [49]. For Northern Ireland as a whole, integrating a software developed for use in acute care community hospital settings, combining real-time antibiotic prescribing and microbiology reports- into primary care could serve as an AMS tool to reduce inappropriate antibiotic prescribing, and overall consumption, thereby mitigating Northern Ireland`s contribution to AMR [50].

A secondary aim of this study was to investigate the impact of the COVID-19 pandemic on antimicrobial prescribing and consumption patterns in GP practices in Northern Ireland. This was achieved via analysis of November 2020, December 2020, and January 2021 BSO open access data, and comparing it to the BSO data in the period prior to the COVID-19 pandemic. Data from April 2020 were not included in the analysis due to the shutdown of services as a result of a national lockdown at the beginning of the pandemic [51].

Unexpectedly, there was no significant difference between the antimicrobial prescribing and consumption patterns in GP practices during the COVID-19 pandemic when compared to the pre-pandemic period. This contrasts with a previous study that found that because of the difficulty in differentiating between COVID-19 and bacterial pneumonia at the beginning of the pandemic, due to the similarity in symptoms, antibiotics were inappropriately prescribed for the treatment of COVID-19 [21]. Another study was consistent with the findings of this study found an increase in COVID-19 cases in secondary care, with a higher rate of antimicrobials prescribed, despite a low rate of bacterial co-infection in patients positive for COVID-19 infection [23]. Although both studies agree with the empirical prescribing guidelines used by GPs based on signs and symptoms [28], as well as the expected decrease in GP visits during the COVID-19 pandemic, considering the restriction on access to healthcare services (NHS Digital 2020) and thus reduced access to AST and COVID-19 testing [24], they fail to explain the lack of significance in antimicrobial prescribing found in this study.

GP face-to-face appointments in Northern Ireland decreased by 19% at the beginning of the COVID-19 pandemic compared to the previous year. However, this reduction can be explained by the time GPs spend working at COVID-19 centres, which equates to equated to a 19% reduction in surgery working time [52]; thus, the lack of significant change in antimicrobial prescribing pre- and midst-COVID-19 pandemic, can be attributed to the reduced time GPs spent in their surgeries. Additionally, while there was an overall reduction in face-to-face appointments, it is important to note that this does not equate to a reduction in total appointments; GP surgeries simply operated differently in response to the pandemic, replacing in-person appointments with telephone and/or online consultations [53]. Furthermore, 35% of patients sought advice from community pharmacies instead of GPs in November 2020 due to COVID-19 measures [54], potentially freeing up appointments for more serious cases. Measures implemented throughout the pandemic, such as working from home, social distancing, and more frequent hand washing are thought to have not only helped reduce the spread of COVID-19 but as well as other infections, in general, across the population. A study of primary care in England found a 39.13% reduction in prescribing for respiratory tract infections between March and September 2020, compared to the previous year [24]. These social distancing and hygiene measures were UK-wide; therefore, a reduction in infections meant fewer patients were seeing their GP for an antibiotic.; This along with the already declining trend in antimicrobial prescribing prior to the COVID-19 pandemic [22] resulted in a decrease in antimicrobial prescribing. The decline in antibacterial prescribing is thought to have been counteracted by the empirical prescribing for COVID-19 infections due to the similarity of symptoms with other respiratory tract infections. Therefore, overall this resulted in no significant difference between antibacterial prescribing pre- and midst-COVID-19 pandemic.

Despite no overall significant difference between antimicrobial prescribing pre- and midst-COVID-19 pandemic, individual classes of antibiotics showed varied prescribing patterns. Carbapenems and glycopeptides increased substantially during the COVID-19 pandemic with, +96.88% and +69.34% change, respectively. Carbapenems are broad-spectrum antibiotics that are reserved as a last resort antibiotic against Gram-negative and Gram-positive drug-resistant infections [55]. The increased use of carbapenems during the COVID-19 pandemic in this study correlates with a study carried out in secondary care ICU [56]. However, more research is required to understand the reasoning behind its increased use in primary care during the COVID-19 pandemic. Glycopeptides, although antibacterial against Gram-positive bacteria, were found to have significant activity against SARS-COV2, interfering with its replication. Therefore, glycopeptides, such as teicoplanin, were repurposed for the treatment of COVID-19 [57], which might explain the considerable increase in its use during the COVID-19 pandemic.

As previously stated, prior to the COVID-19 pandemic, penicillins, tetracyclines, and macrolides were the most prescribed classes of antibiotics in Northern Ireland. However, during the COVID-19 pandemic all three classes decreased in prescribing, −86.37%, −10.63%, and −42.16% change, respectively. This differs from a study in the Republic of Srpska where the top three antibiotics repurposed and prescribed during the pandemic were amoxicillin/clavulanic acid, doxycycline, and azithromycin [58]. Again, this study looked at DDD and focused on COVID-19 outpatients. Therefore, a study based on DDD per population may have been a more accurate way of comparison between Northern Ireland and the rest of the world. However, a reduction in consumption of these antibiotics during COVID-19 is a step in the right direction in terms of Northern Ireland’s contribution to AMR, specifically with the macrolide antibiotic azithromycin, which has increased the likelihood of developing resistance as a Watch group antibiotic.

To the best of the investigator’s knowledge, this is the first study to explore antibacterial consumption among GP federations in Northern Ireland and compare antimicrobial prescribing rates against the WHO’s AWaRe Classification future targets. Limitations of this study include the lack of adjustment for patient numbers per federation and socio-demographic factors, which could have provided deeper insights into prescribing patterns. Furthermore, due to data restrictions and time constraints, comparisons using Defined Daily Dose (DDD) were not feasible. Additionally, only prescribing data were collected, not actual consumption data.

## 4. Materials and Methods

### 4.1. Study Design

A retrospective, cross-sectional quantitative study was designed to measure, analyse, and evaluate the antimicrobial prescribing patterns within GP practices in Northern Ireland. This study was conducted nationally within all GP practices in Northern Ireland using the open access BSO data to retrospectively collect the antimicrobials prescribed for a defined period; April 2019, November 2019, December 2019, November 2020, December 2020, and January 2021.

Data were retrospectively collected through the open access BSO data. The BSO department was emailed to enquire specifically for the following months: March 2019–January 2021 inclusive to extract and analyse antimicrobial prescribing patterns in primary care in Northern Ireland during a specified period of time.

Data collection forms were designed and developed by the investigators to organise and categorise data received from the BSO. All data collected were completely anonymous with no identifiers gathered. The data collection forms were designed to enable the assessment and analysis of all necessary information. Data such as GP practice code, year, month, name of antimicrobial product, class of antimicrobial, presentation, strength, total quantity, and total amount of antimicrobial in grams were collected. This comprised the quantity of individual drugs prescribed per month, for each GP practice in Northern Ireland.

Open access BSO GP prescribing data for April 2019, November 2019, December 2019, November 2020, December 2020, and January 2021 were also included in the study, to comprise different seasons.

### 4.2. Inclusion Criteria

- Antimicrobials prescribed by a GP, nurse or other non-medical prescriber attached to a GP practice in Northern Ireland during the months of April 2019, November 2019, December 2019, November 2020, December 2020, and January 2021;
- Antimicrobial prescriptions that were subsequently taken to a community pharmacy and dispensed;
- Antimicrobials that were ordered on a stock form;
- Antimicrobials prescribed for all age brackets;
- All antimicrobial dosage forms for each antimicrobial drug.

### 4.3. Exclusion Criteria

- Antimicrobials prescribed within secondary care;
- Antimicrobials prescribed outside the defined period of the study as stated within the inclusion;
- Antimicrobial prescriptions that were not subsequently taken to a community pharmacy and dispensed;
- Hospice items;
- Private prescriptions.

### 4.4. Method of Statistical Analysis

GP-prescribed antimicrobials were collected, extracted and categorised from the open access BSO COMPASS report to Microsoft^®^ Excel^®^ for Microsoft 365 MSO (Version 2311). Using IBM SPSS Statistics (Version 28.0.1.1 (15)), data were first tested for normality using the Kolmogorov–Smirnov test before being quantitatively analysed using a one-sample *t*-test to determine statistical significance between overall antibiotic prescribing across GP federations. A comparison of the amount of the three main antibiotics prescribed across GP federations was statistically analysed using two-way ANOVA to test statistical significance. Data were then presented as mean (±standard error of the mean).

Data collected for comparison of prescribing pre- and midst-COVID-19 pandemic were tested for normality using the Kolmogorov–Smirnov test. However, the data did not follow normality therefore was log transformed to conform to normal distribution. It was then quantitatively analysed using an unpaired *t*-test on GraphPad Prism (Version 10.1.0 (264)). Additionally, the chi-square test was also employed in the analysis.

### 4.5. Ethical Approval

Ethical approval was not required for this study as it utilised anonymous, retrospective, open access BSO data which is publicly available.

## 5. Conclusions

Northern Ireland’s antimicrobial prescribing rates in GP practices remain higher than those in the rest of the UK, despite achieving the WHO target of having at least 60% of antibiotics prescribed from the Access group. Although Northern Ireland has achieved this target, overprescribing of antibiotics, regardless of AWaRe classification, contributes to AMR due to inappropriate prescribing. Although antibiotic prescribing rates in Northern Ireland are on the decline, there was no significant difference amongst GP federations pre- and midst- the COVID-19 pandemic. This suggests that enhanced strategies are needed, such as increased communication between colleagues, increased consultation time with patients, and support resources within GP surgeries. These measures are crucial for advancing AMS amongst the GP federations, and thus reduce both antimicrobial consumption and resistance.

Future research should adjust for socio-demographic factors and report antibiotic consumption in Defined Daily Doses (DDD) to gain a comprehensive understanding of Northern Ireland’s role in AMR and identify effective reduction strategies. Additionally, exploring the perspectives of GPs and GPPs within the practice teams could provide valuable insights into necessary support changes for antibiotic prescribing. Such efforts could significantly impact Northern Ireland’s AMR situation and contribute to mitigating the global threat, ultimately benefiting future generations.

## Figures and Tables

**Table 1:** The amount of each antibiotic class prescribed in GP surgeries across Northern Ireland throughout the duration of the study.

Antibiotic Classification	April-19	November-19	December-19	Total (grams)	Percentage
Aminoglycosides	0.80	17.86	15.96	34.62	0.00%
Amphenicol	4.00	10.00	1.00	15.00	0.00%
Antimycobacterial	1393.70	1301.00	1202.60	3897.30	0.12%
Beta-lactam/Beta-lactamase inhibitors	66,787.50	69,896.21	78,732.02	215,415.73	6.80%
Beta-lactam/Beta-lactamase inhibitors antipseudomonal	189.00	-	-	189.00	0.01%
Carbapenems	-	-	8.00	8.00	0.00%
First-generation cephalosporins	39,951.50	40,391.00	43,340.75	123,683.25	3.90%
Fluoroquinolones	17,442.70	16,457.05	18,192.95	52,092.70	1.64%
Fusidane	114.50	232.75	83.00	430.25	0.01%
Glycopeptides	20.25	29.05	58.00	107.30	0.00%
Imidazoles	13,820.74	13,318.24	13,004.60	40,143.58	1.27%
Lincosamides	2268.60	2200.80	2064.60	6534.00	0.21%
Macrolides	91,898.39	101,155.63	108,854.30	301,908.32	9.53%
Nitro-furan derivatives	10,635.64	10,847.75	11,235.98	32,719.37	1.03%
Oxazolidinones	16.80	6.00	51.60	74.40	0.00%
Penicillins	531,446.15	655,415.20	707,796.90	1,894,658.25	59.79%
Phosphonics	135.00	270.00	243.00	648.00	0.02%
Rifamycins	14,010.45	15,289.30	15,366.50	44,666.25	1.41%
Rifamycins + antimycobacterial	953.60	1666.40	1318.50	3938.50	0.12%
Second-generation cephalosporins	810.33	790.70	1015.95	2616.98	0.08%
Steroid antibiotics	62.50	47.50	-	110.00	0.00%
Sulfonamides	11,420.88	11,717.76	13,082.88	36,221.52	1.14%
Sulfones	450.60	460.50	403.90	1315.00	0.04%
Tetracyclines	115,584.95	109,268.74	113,494.76	338,348.46	10.68%
Third generation cephalosporins	74.00	85.70	146.60	306.30	0.01%
Trimethoprim-derivates	22,766.35	23,209.30	22,717.66	68,693.31	2.17%
Subtotal (grams)	942,258.93	1,074,084.44	1,152,432.01	3,168,775.38	100%

**Table 2:** Amount of Access, Watch and Reserve group antibiotics prescribed in GP surgeries across Northern Ireland throughout the duration of the study.

AWaRe Classification	Amount (g)	Percentage
**ACCESS**	2,506,756.21	79.35%
**WATCH**	652,113.72	20.64%
**RESERVE**	74.40	0.00%
	**3,158,944.33**	**100.00%**

**Table 3:** The total am ount of penicillins, tetracyclines, andm acrolides prescribed and dispensed in GP federationsacross Northern Ireland throughout duration of study.

Total Amount (g) of Antibiotic Class Prescribed			
GP Federation	Penicillins	Tetracyclines	Macrolides
Antrim Ballymena	129,710.10	22,086.71	17,792.24
Ards	72,652.55	14,119.61	12,113.89
Armagh & Dungannon	117,887.80	17,948.50	19,629.00
Causeway	102,822.35	21,411.17	15,748.15
Craigavon	120,164.60	18,176.18	19,659.99
Derry	210,782.50	35,460.46	30,704.76
Down	73,314.70	13,791.18	12,108.70
East Antrim	151,976.35	30,666.98	23,779.80
East Belfast	105,696.60	20,475.78	15,863.75
Lisburn	80,267.75	16,658.10	13,944.91
Mid-Ulster	80,612.20	12,227.19	9641.84
Newry & District	150,399.00	22,860.04	24,875.25
North Belfast	112,155.35	20,639.17	19,458.81
North Down	63,978.10	14,970.18	11,926.69
South Belfast	85,056.80	20,116.01	15,006.32
Southwest	139,829.90	16,669.01	24,482.82
West Belfast	97,378.60	20,072.18	15,198.36

## Data Availability

Data will be made available upon request.
